# Synthesis of Metal/SU-8 Nanocomposites through Photoreduction on SU-8 Substrates

**DOI:** 10.3390/nano13111784

**Published:** 2023-06-01

**Authors:** Yan-Jun Huang, Wen-Huei Chang, Yi-Jui Chen, Chun-Hung Lin

**Affiliations:** 1 Department of Photonics, National Cheng Kung University, Tainan 70101, Taiwan; 2 Department of Applied Chemistry, National Pingtung University, Pingtung 90003, Taiwan

**Keywords:** nanocomposite, metal nanoparticles, photoreduction, plasmonic structure, nanofabrication, SU-8, antibacterial, color filter

## Abstract

The paper presents a simple, fast, and cost-effective method for creating metal/SU-8 nanocomposites by applying a metal precursor drop onto the surface or nanostructure of SU-8 and exposing it to UV light. No pre-mixing of the metal precursor with the SU-8 polymer or pre-synthesis of metal nanoparticles is required. A TEM analysis was conducted to confirm the composition and depth distribution of the silver nanoparticles, which penetrate the SU-8 film and uniformly form the Ag/SU-8 nanocomposites. The antibacterial properties of the nanocomposites were evaluated. Moreover, a composite surface with a top layer of gold nanodisks and a bottom layer of Ag/SU-8 nanocomposites was produced using the same photoreduction process with gold and silver precursors, respectively. The reduction parameters can be manipulated to customize the color and spectrum of various composite surfaces.

## 1. Introduction

Nanoparticles of precious metals, such as silver and gold, have gained significant interest in the research community due to their unique properties [1]. These properties enable a wide range of applications across various scientific fields. For instance, adjusting the distribution, size, and shape of nanoparticles can manipulate the optical phenomena of localized surface plasmon resonance (LSPR), which differs from the characteristics of bulk metals [2] and has led to the development of applications in optical devices [3] and biomedical sensing [4]. Among the precious metal nanoparticles, silver nanoparticles (AgNPs) have shown great promise in the fields of sensing and catalysis due to their compatibility [5] and antibacterial properties. These properties make AgNPs ideal for use in biomedical and food packaging applications [6,7].

Incorporating nanoparticles into polymer media can offer diverse applications and uses, while maintaining their physical properties through packaging. Various standard patterning methods, including optical lithography [8], electron-beam lithography [9], and nanoimprinting [10], can be used to control the distribution and size range of nanoparticles, leading to the development of new patterning and material applications [11]. However, achieving an even distribution of nanoparticles in the polymer medium through the common metal deposition methods used in semiconductor processes can be challenging [12]. The recent research has focused on two mainstream production methods for mixing the composites. The first method involves pre-mixing the metal precursor with the polymer to form a stable colloid solution, followed by the reduction using electron-beam, UV exposure, or chemical reduction [9,13,14]. The second method involves mixing pre-synthesized nanoparticles with the polymer or growing them layer by layer [15,16]. The resulting nano-polymer composites can be used in various applications in the fields of electronics, optoelectronics, and biomedicine, including photopatterned electrodes [17], plasmonic sensor [8,18], and Raman sensing substrates [15]. However, these methods often require the preparation of nano-precursor solutions or the pre-synthesis of nanoparticles. The process can be cumbersome and time-consuming and may not ensure the long-term stability of storage properties or allow for post-adjustment. Additionally, care must be taken to prevent the aggregation of nanoparticles during growth [19]. Moreover, the use of electron beams and lasers for growth requires significant time and cost, especially in mass production.

SU-8 photoresist is a widely used negative-tone chemically amplified photoresist due to its heat and acid/base resistance [20], as well as its excellent mechanical properties. It finds extensive applications in the fields of microelectromechanical systems (MEMS) and microfluidics [21]. Its high compatibility with biological systems [22] also makes it suitable for use in biosensors. Additionally, its high transparency in the visible light range renders it applicable in optics [23]. SU-8 is typically spin-coated on wafers using standard processes and then undergoes patterning. Its characteristic of generating a large number of free radicals under UV exposure and elevated temperature also makes it an ideal material for reducing metal precursors within its structure via optical lithography [24,25,26]. For instance, Tan et al. made Au/SU-8 nanocomposites through the photoreduction of a gold precursor and SU-8 mixture for electrodes and grating applications [24]. Fischer et al. fabricated Ag/SU-8 nanocomposites through the photoreduction of a silver precursor and SU-8 mixture and explored their localized surface plasmon resonance (LSPR) response [25]. However, when mixing with the nanoparticles in solution form, the loading effect in the solution influences the spin-coating quality of the film and nanoparticle distribution inside the film.

In our previous study, we demonstrated the direct synthesis of a monolayer of gold nanoparticles (AuNPs) on the surface of SU-8 under UV exposure [26]. In this study, we would like to investigate the SU-8 photoreduction behavior on the silver precursor. A drop of the silver precursor was simply applied on the SU-8 surface, followed by UV exposure, without the need for mixing the precursor and SU-8 in advance. Additional photoinitiators (PIs) were added to SU-8 to test their ability to accelerate the reduction efficiency. The combination of the photoreduction of the gold and silver precursors on the same SU-8 surface, respectively, was also studied. AgNPs and AuNPs have distinct LSPR ranges in the visible spectrum, providing significant potential for color tuning in color filter applications through tailoring of their LSPR. Furthermore, the antibacterial ability of the reduced AgNPs was tested.

## 2. Materials and Methods

### 2.1. Chemicals and Materials

Perfluoropolyether (PFPE)-urethane dimethacrylate (Fluorolink MD700) was obtained from Solvay Specialty Polymers (Bollate, Italy). Additionally, 2,2-dimethoxy-2-phenylacetophenone and triarylsulfonium hexafluoroantimonate salts (mixed, 50 wt.% in propylene carbonate) were obtained from Sigma-Aldrich (St. Louis, MO, USA). SU-8 3025 was purchased from Kayaku Advanced Materials (Westborough, MA, USA). Poly(methyl methacrylate (PMMA, molecular weight: 35 k), silver nitrate (AgNO_3_), gold (III) chloride trihydrate (HAuCl_4_·3H_2_O), cyclopentanone, and 1H,1H,2H,2H-perfluorodecyltrichlorosilane (F_13_-TCS) were purchased from Alfa Aesar (Ward Hill, MA, USA). Additionally, 1,1,2-Trichloro-1,2,2-trifluoroethane was purchased from Grand Chemical Co. (Miaoli, Taiwan). The solvents and chemicals were used without further purification.

### 2.2. Synthesis of Ag/SU-8 Nanocomposites by Photoreduction

A glass substrate (1.25 cm × 1.25 cm) was cleaned in acetone and isopropanol with an ultrasonic bath for 30 min and dried with a nitrogen flow. The SU-8 solution (20 μL SU-8 3025/cyclopentanone at a weight ratio of 1:8) was then spin-coated on the glass substrate at 500 rpm for 5 s and 2500 rpm for 25 s. The SU-8 film was then subjected to a softbake process at 95 °C for 10 min, resulting in a film thickness of approximately 300 nm. The SU-8 film was initially cured with an i-line UV mercury lamp (λ = 365 nm, 150 mW/cm^2^) for 1 min. A drop of AgNO_3_ aqueous solution (80 μL) was then dripped onto the SU-8 surface using a quantitative pipette. The same mercury lamp was employed again to expose the photoresist for 18 min, leading to the photochemical formation of AgNPs in the resist film. The process can be repeated for additional cycles after washing the sample with DI water.

### 2.3. Nanoimprint of SU-8 Nanopillar Arrays [26]

Nanoimprint lithography was employed to pattern SU-8 nanopillar arrays. An SU-8 resist film was prepared as described in Section 2.2. A PFPE working mold was replicated from a silicon master mold with anti-sticking treatment through the vapor deposition of F_13_-TCS [27]. PFPE possesses a lower surface energy, making it easier to demold from the imprinted SU-8 polymers. A mixture of Fluorolink MD700 and its photoinitiator (2,2-dimethoxy-2-phenylacetophenone, 1 wt%) was prepared and poured onto the master mold. The solvent used for PI was 1,1,2-Trichloro-1,2,2-trifluoroethane. The sample was then placed in a vacuum and cured under UV light. After the curing, PFPE was released from the silicon master mold. Subsequently, the PFPE mold was placed on top of the SU-8 film, and the nanoimprint process was carried out using our home-built nanoimprint platform. The imprinting pressure was set at 3 bar for 10 min while maintaining a constant temperature of 80 °C. After cooling, the nanoimprinted SU-8 nanostructures were obtained by removing the mold from the resist. The process of the photochemical formation of AuNPs on SU-8 nanopillar arrays was the same as that used for an SU-8 film.

### 2.4. Growth of Gold Nanodisks on SU-8 through Photoreduction

The growth of gold nanodisks was achieved through the selective growth of patterned monolayer AuNPs via photoreduction [28]. To confine the area for AuNP production, a PMMA nanohole array mask was applied onto the SU-8 surface using a nanotransfer printing process. After exposing a drop of HAuCl_4_ over the PMMA-masked SU-8 surface to UV light, AuNPs formed on the uncovered SU-8 surface. A gold nanodisk array was obtained after three cycles of photoreduction.

### 2.5. Characterization

Scanning electron microscopy (SEM) images were obtained using a field emission scanning electron microscope (JEOL 6340F, Tokyo, Japan), while transmission electron microscopy (TEM) samples were prepared using a focused ion beam system (FEI Helios G3CX, Thermo Fisher Scientific, Waltham, MA, USA). TEM brightfield images and energy scattering spectroscopy (EDS) were acquired using JEOL JEM-2100F CS STEM (Tokyo, Japan). The transmission spectra were measured using a miniature UV-VIS spectrometer (Model: BLK-CSR-SR, StellarNet Inc., Tampa, FL, USA), and a tungsten halogen light source (SL1-FILTER, StellarNet Inc., Tampa, FL, USA, and SLS301, Thorlabs Inc., Newton, NJ, USA). X-ray photoelectron spectroscopy (XPS) was carried out using PHI 5000 VersaProbe (ULVAC-PHI, Kanagawa, Japan). The CIE 1931 color space chromaticity diagrams were created by converting and mapping the measured spectra.

### 2.6. Antibacterial Screening Test

The experimental procedure for preparing the filter paper with the Ag/SU-8 nanocomposites used for the antibacterial screening test was carried out as follows: 1. apply 20 μL of SU-8 onto the filter paper (the sample). 2. Position the sample on a hotplate and set the temperature to 95 °C. Heat the sample to remove surface solvents and allow it to remain at this temperature for 10 min. 3. Transfer the sample to a mercury lamp exposure box for light pre-curing. Expose the sample to light for 5 min, then carefully remove it from the exposure box. 4. Apply 90 μL of AgNO_3_ solution onto the surface of the sample. Place the sample back into the mercury lamp exposure box to initiate the photoreduction process for AgNPs. Allow the sample to be exposed to light for 18 min, then remove it from the exposure box. 5. Clean the surface of the sample by rinsing it with DI water. Use a nitrogen gun to dry the sample thoroughly. 6. Optionally, repeat steps 4 and 5 to increase the number of reduction cycles as desired. 7. Once again, position the sample on the hotplate and set the temperature to 95 °C. Heat the sample to eliminate any remaining surface moisture and allow it to stay at this temperature for 10 min. The Gram-positive Staphylococcus aureus (BCRC 10451) and Gram-negative Escherichia coli (BCRC 11634 and NCTC 11954) strains were utilized in this study. BCRC 10451 and NCTC 11954 are antibiotic-resistant strains that can withstand penicillin and streptomycin treatments. The strains were cultivated in nutrient broth medium (g/L: Tryptone 10; yeast extract 5; NaCl 10; pH 7.5) at 37 °C with continuous shaking at 120 rpm. The bacterial cell suspensions were diluted with sterile water to achieve a final concentration of 10^8^ CFU/mL. For seeding the agar plates, 100 µL of this bacterial suspension was used. Filter paper disks with a diameter of 8 mm, incorporating Ag/SU-8 nanocomposites, were placed onto the surface of the seeded medium. After incubating at 37 °C for 24 h, the zones of inhibition, which indicate areas where bacterial growth is inhibited, were measured. As a control group in this study, filter paper disks without the application of SU-8, but following the previously described experimental procedure, were utilized.

## 3. Results and Discussion

### 3.1. Synthesis and Characterization of Ag/SU-8 Nanocomposites

**Figure 1:**
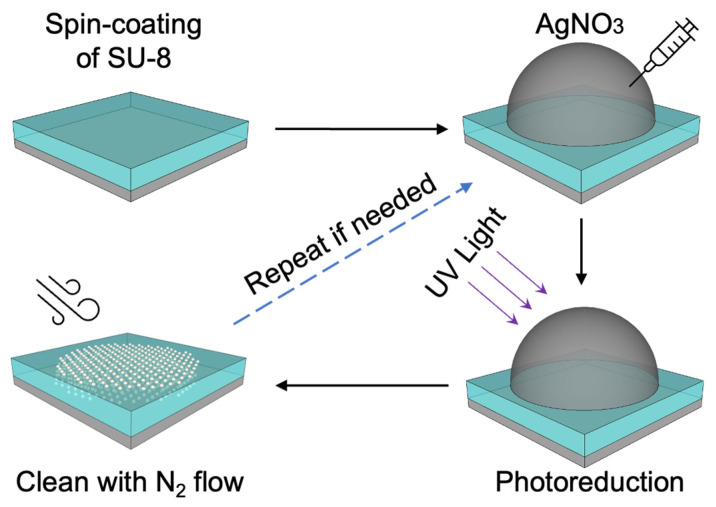
The process flow for synthesizing Ag/SU-8 nanocomposites.

**Figure 2:**
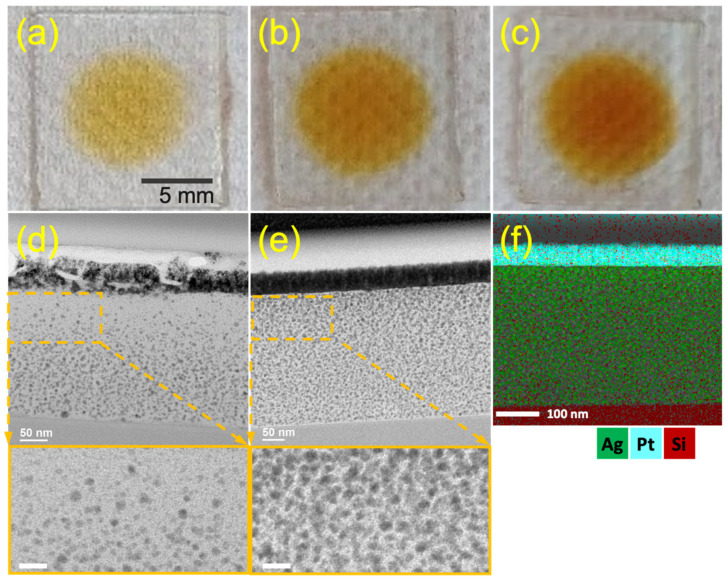
(**a**–**c**) Optical images of the Ag/SU-8 nanocomposites after one, two, and three photoreductions. The concentration of AgNO_3_ was 0.5 mM. (**d**,**e**) Cross-sectional TEM images of the Ag/SU-8 nanocomposites photoreduced from 0.5 and 50 mM AgNO_3_, respectively. (**f**) EDS elemental analysis of Ag/SU-8 nanocomposites photoreduced from 50 mM AgNO_3_. The scale bars in the insets at the bottom of (**d**,**e**) measure 20 nm.

The process of producing Ag/SU-8 nanocomposites is illustrated in Figure 1. A drop of silver precursor (AgNO_3_) was placed on the SU-8 surface, and then, UV exposure was applied to initiate the reduction of AgNPs. The SU-8 film turned yellow and became progressively darker with repeated photoreduction, as seen in Figure 2a–c. The deepening color of the surface from the first to the third photoreduction suggests an increase in the concentration of the produced AgNPs. A TEM observation was conducted to confirm the composition and depth distribution of the AgNPs. Figure 2d,e display the TEM bright field images of the Ag/SU-8 nanocomposites reduced with the silver precursor (0.5 and 50 mM, respectively). In contrast to the reduced AuNPs that form a monolayer on the SU-8 film [26,28], the reduced AgNPs penetrated the SU-8 film and formed the Ag/SU-8 nanocomposites. The molecular weights of silver nitrate and chloroauric acid are approximately 169.87 and 339.79 g/mol, respectively. The large size of chloroauric acid may prevent it from penetrating into SU-8, which could explain why AuNPs grow on the SU-8 surface while AgNPs form inside the SU-8. The AgNPs were extremely small, with a size of less than 10 nm, due to the limited space available for growth within the SU-8 polymer. The AgNPs with 0.5 mM AgNO_3_ tended to distribute towards the bottom side of the SU-8, while the AgNPs with 50 mM AgNO_3_ were evenly and densely distributed throughout the entire area of the SU-8 film. Figure 2f presents the EDS elemental analysis of the same area in Figure 2e. The sample was coated with a platinum layer, which was identified and labeled with green points. The substrate was silicon and marked with red points. The layer on the substrate was SU-8 and the silver element (green points) was uniformly distributed within the SU-8 film.

**Figure 3:**
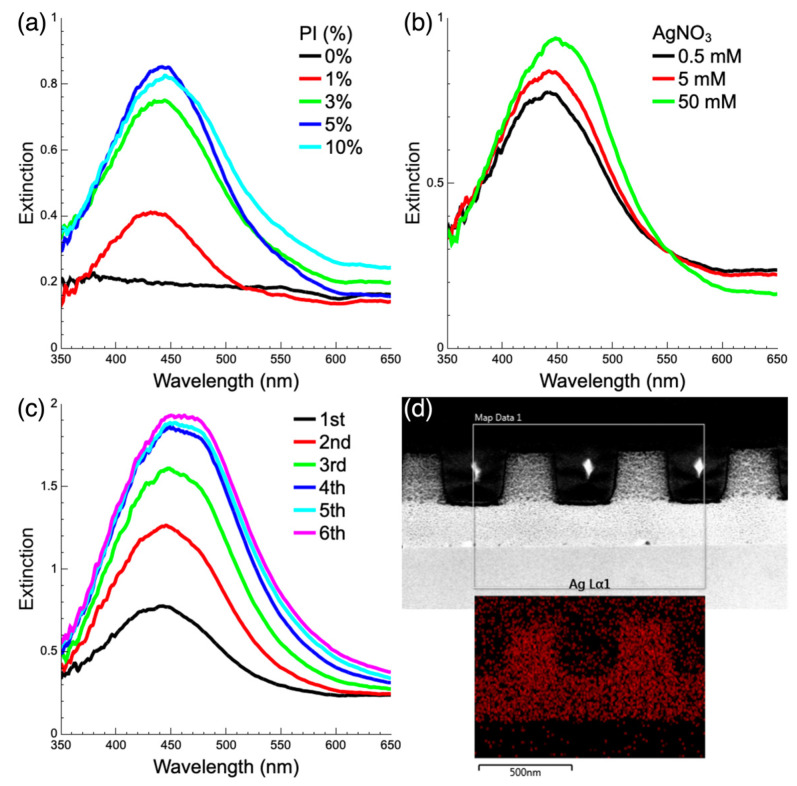
Extinction spectra of the Ag/SU-8 nanocomposites under various experimental conditions. (**a**) PI was added to SU-8 at concentrations ranging from 0 to 10 wt% with a fixed AgNO_3_ concentration of 0.5 mM. (**b**) PI concentration was fixed at 5 wt% with AgNO_3_ concentrations ranging from 0.5 to 50 mM. (**c**) Photoreduction was performed from one to six cycles with fixed concentrations of PI and AgNO_3_ at 5 wt% and 0.5 mM, respectively. (**d**) Cross-sectional TEM image of the Ag/SU-8 nanocomposites photoreduced from 50 mM AgNO_3_ on SU-8 nanopillars. The bottom inset shows EDS elemental analysis of the silver element.

The extinction spectra of Ag/SU-8 nanocomposites are displayed in Figure 3. A prominent absorption peak is observed at a wavelength of 440 nm, corresponding to the LSPR peak of reduced AgNPs. The effect of photoreduction parameters on AgNPs is evident from the extinction spectra. Figure 3a illustrates the impact of varying additional PI concentrations on photoreduction. The PI used for SU-8 polymerization is triarylsulfonium hexafluoroantimonate salt, a photoacid generator. Diluted SU-8 (SU-8 3025/cyclopentanone at a weight ratio of 1:8) was mixed with varying additional PI concentrations ranging from 0 to 10 wt%. The SU-8 without added PI (0 wt%) is the diluted commercial SU-8 that already contains a certain amount of PI. As the amount of added PI increases, the photoacid generation rate during UV exposure also increases, leading to the production of more free radicals required for reduction [26]. The most notable difference is observed between SU-8 without added PI and SU-8 with 1 wt% added PI. All samples with added PI exhibit a 440 nm absorption peak corresponding to AgNP LSPR after the first reduction, while the original SU-8 sample shows no significant absorption peak. The original SU-8 requires multiple reductions before its reduction effect approaches that of the PI-added sample (see Figure S1), indicating that adding PI improves reduction efficiency. As the amount of PI increases, the intensity of the absorption peak also increases, reaching its maximum at 5 wt% PI. To maintain consistency in subsequent experiments and facilitate observation of differences in other parameter effects, all subsequent experiments used SU-8 with 5 wt% added PI. Figure 3b shows reduction results at various AgNO_3_ concentrations. As PI concentration increases, the LSPR absorption also increases, consistent with the increase in AgNP density shown in the TEM images of Figure 2a,b. Figure 3c displays the spectra of samples subjected to multiple photoreduction cycles. As the number of photoreduction cycles increases, the corresponding absorption peak intensity strengthens and the bandwidth widens. After the fourth reduction, however, the extinction peak does not exhibit a significant increase. The photoreduction process can also be performed directly on SU-8 nanostructures, resulting in the formation of Ag/SU-8 nanocomposites with a nanostructured shape. First, a nanopillar array with a diameter of 300 nm, a period of 600 nm, and a height of 300 nm was patterned through nanoimprint lithography [26]. Then, a drop of AgNO_3_ solution was applied to the surface of the SU-8 nanopillar array and exposed to UV light. Figure 3d shows a cross-sectional TEM image of 50 mM AgNO_3_ photoreduced in a SU-8 nanopillar array with 2 reduction cycles. The AgNPs within the SU-8 nanopillars were identified through EDS elemental analysis, as shown in the bottom inset of Figure 3d. The AgNPs were uniformly distributed within the SU-8 nanopillars.

### 3.2. Composite Surface Composed of Gold Nanodisks and Ag/SU-8 Nanocomposites

**Figure 4:**
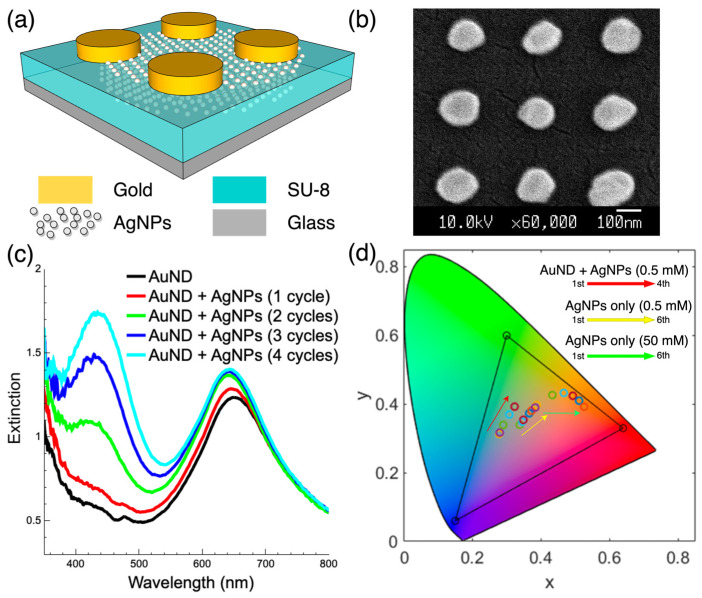
(**a**) The schematic of the composite surface made up of gold nanodisks and Ag/SU-8 nanocomposites. (**b**) The top-view SEM image of the composite surface. (**c**) The measured extinction spectra of the composite surface with the reduction in AgNPs from 0 to 4 cycles. (**d**) The CIE 1931 color space calculated from the measured spectra of the various Ag/SU-8 nanocomposites and composite surfaces.

In our previous study, we demonstrated the selective growth of patterned monolayer AuNPs on SU-8 surfaces [28]. Here, we fabricated a composite surface composed of a top gold nanodisk array and bottom Ag/SU-8 nanocomposites, as shown in Figure 4a, using the same photoreduction process. To create the composite surface, we introduced a PMMA nanohole array mask on the SU-8 surface using a residual layer-free nanotransfer printing process. After three cycles of HAuCl_4_ photoreduction, a gold nanodisk array was achieved. The same reduction process with 1 to 4 cycles was then applied on the same SU-8 surface with the silver precursor of AgNO_3_. The SEM image of the composite surface is shown in Figure 4b. The top gold nanodisks had a diameter of approximately 150 nm, a period of 300 nm, and a height of approximately 100 nm. The AgNPs were inside the SU-8 film and were, therefore, invisible in this image. Figure 4c shows the measured extinction spectra of the composite surfaces with the reduction in AgNPs from 0 to 4 cycles. With the incorporation of AgNPs, the absorption at a wavelength of approximately 650 nm caused by the gold nanodisk array increased slightly, accompanied by slight blue shifts. The absorption at 435 nm wavelength caused by AgNPs increased with the reduction cycles. Figure 4d shows the CIE 1931 color space [29] calculated from the measured transmission spectra of the various Ag/SU-8 nanocomposites and composite surfaces. The triangle in the chromaticity diagram represents the area of the sRGB color space. For the composite surface (gold nanodisks + AgNPs), the color goes from light blue to light green (the red arrow trajectory with reduction cycles from one to four). For the AgNPs-only surface, the color goes from light purple to orange-red (the yellow arrow trajectory with 0.5 mM AgNO_3_ from one to six reduction cycles, and then the green arrow trajectory with 50 mM AgNO_3_ from one to six reduction cycles). By combining the reduction of AuNPs and AgNPs using the same photoreduction system, the composite surfaces can be fabricated in an inexpensive and convenient way. We can adjust the color and spectrum in three different dimensions: 1. adjusting AgNP photoreduction parameters; 2. adjusting geometric shapes of gold nanostructures; 3. the tuning between monolayer AuNPs and bulk gold nanostructures through photoreduction cycles. Compared to the fabrication of metasurface-based color filters [30,31,32], the current approach can achieve similar results with relatively few semiconductor equipment and process steps, avoiding a large amount of expensive and time-consuming e-beam writing time.

### 3.3. Antibacterial Ability of Ag/SU-8 Nanocomposites

**Figure 5:**
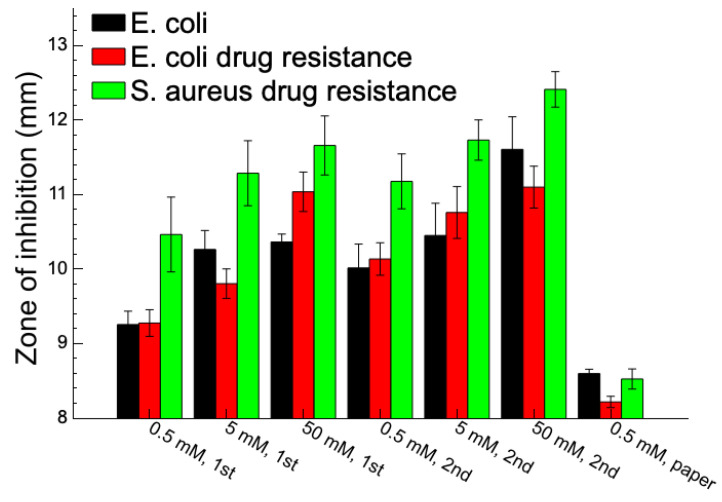
Antibacterial inhibition zones of various Ag/SU-8 nanocomposites against antibiotic-sensitive Escherichia coli, antibiotic-resistant Escherichia coli, and antibiotic-sensitive Staphylococcus aureus.

AgNPs are shown to exert significant inhibitory activity against a broad spectrum of bacteria. The antibacterial activity of Ag/SU-8 nanocomposites was studied using the disk diffusion method. The control group consisted of standard antibiotics, such as Penicillin G and Streptomycin, as well as distilled water and SU-8. Our findings revealed that the Ag/SU-8 nanocomposites exhibited efficient antibacterial activity against both antibiotic-sensitive and antibiotic-resistant strains, as shown in Figure 5. The inhibition zones of Ag/SU-8 nanocomposite against antibiotic-sensitive *E. coli* were 9.3 ± 0.2, 10.3 ± 0.3, and 10.4 ± 0.1 mm at 0.5, 5, and 50 mM of AgNO_3_ on SU-8 with one photoreduction cycle. Increasing the photoreduction cycles to two resulted in larger inhibition zones of 10.0 ± 0.3, 10.5 ± 0.4, and 11.6 ± 0.4 mm at 0.5, 5, and 50 mM of AgNO_3_ on SU-8. Similar trends were observed in the other two antibiotic-resistant strains tested. The inhibition zones of Ag/SU-8 nanocomposite against antibiotic-resistant E. coli were 9.3 ± 0.2, 9.8 ± 0.2, and 11.0 ± 0.3 mm at 0.5, 5, and 50 mM of AgNO_3_ on SU-8 with one photoreduction cycle. With two photoreduction cycles, the inhibition zones increased to 10.1 ± 0.2, 10.8 ± 0.3, and 11.1 ± 0.3 mm at 0.5, 5, and 50 mM of AgNO_3_ on SU-8. For another antibiotic-resistant, Gram-positive Staphylococcus aureus strain, the inhibition zones of Ag/SU-8 nanocomposite were 10.5 ± 0.5, 11.3 ± 0.4, and 11.7 ± 0.4 mm at 0.5, 5, and 50 mM of AgNO_3_ on SU-8 with one photoreduction cycle. Increasing the photoreduction cycles to two resulted in larger inhibition zones of 11.2 ± 0.4, 11.7 ± 0.3, and 12.4 ± 0.2 mm at 0.5, 5, and 50 mM of AgNO_3_ on SU-8. The results indicated that the concentration of Ag/SU-8 nanocomposites and the number of photoreduction cycles increased the AgNP density and enlarged the inhibition zones. The control group, consisting of filter paper disks without SU-8 application but treated with AgNO_3_ photoreduction, demonstrated an inhibition zone ranging from approximately 8.2 to 8.6 mm. This result signifies that SU-8 effectively enhanced the photoreduction efficiency of AgNPs and its antibacterial effect. The data representing this control group were presented as 0.5 mM paper on the rightmost side of Figure 5. There are several possible reasons why Ag/SU-8 nanocomposites may be more efficient against antibiotic-resistant strains compared to antibiotics: First, a different mechanism of action: antibiotics typically target specific cellular components or processes, such as cell wall synthesis or protein synthesis. Bacteria can develop resistance to antibiotics by evolving mechanisms to bypass or neutralize these targets. In contrast, AgNPs are believed to work through a combination of mechanisms, including disrupting the bacterial cell membrane, generating reactive oxygen species, and interfering with cellular signaling pathways. This makes it harder for bacteria to develop resistance to AgNPs, as they would need to evolve multiple mechanisms to counteract these different modes of action. Second, broad-spectrum activity: AgNPs were shown to have broad-spectrum antibacterial activity, meaning they can kill a wide range of bacterial species. This contrasts with antibiotics, which are often more specific to certain types of bacteria. Because AgNPs are effective against a broad range of bacteria, including antibiotic-resistant strains, they may be more useful in treating infections caused by multiple bacterial species or strains. Third, the lower likelihood of resistance: overuse and misuse of antibiotics has led to the emergence of antibiotic-resistant bacteria, which can render antibiotics ineffective. Because AgNPs have multiple modes of action, it may be harder for bacteria to develop resistance to them compared to antibiotics. Additionally, some studies suggest that prolonged exposure to AgNPs may select for resistant strains, but the likelihood of this happening may be lower than with antibiotics. Overall, AgNPs may be more efficient against antibiotic-resistant strains of bacteria because of their different mode of action, broad-spectrum activity, and lower likelihood of resistance. However, more research is needed to fully understand the mechanisms underlying the antibacterial activity of AgNPs and their potential for long-term use in treating bacterial infections.

## 4. Conclusions

This study demonstrated the synthesis of AgNPs via the photoreduction of silver nitrate on SU-8. In contrast to the photoreduction of chloroauric acid, which produces monolayer AuNPs on the SU-8 surface, silver nitrate penetrates into SU-8 and forms Ag/SU-8 nanocomposites. Simply a drop of the silver precursor on the SU-8 surface is followed by UV exposure without the need of premixing the precursor and SU-8 or synthesizing AgNPs in advance. The impact of photoreduction parameters on AgNPs is evident from the measured extinction spectra, which vary with PI concentrations, AgNO_3_ concentrations, and the number of photoreduction cycles. The photoreduction process can be directly applied to SU-8 nanostructures, resulting in the formation of Ag/SU-8 nanocomposites with a nanostructured shape, which is very appealing for nanodevice fabrication. Using the same photoreduction process, a composite surface consisting of a top layer of gold nanodisks and a bottom layer of Ag/SU-8 nanocomposites was fabricated. By adjusting the silver precursor concentration, photoreduction parameters, and geometry of the gold nanostructures, the transmission spectra of the composite surfaces can be customized, demonstrating their potential for color tuning in color filter applications. The Ag/SU-8 nanocomposites also exhibit antibacterial activity against two Gram-negative Escherichia coli and one Gram-positive Staphylococcus aureus. They were shown to be more effective against antibiotic-resistant strains. Increasing the AgNPs density in the nanocomposites can enhance their inhibitory activity against bacteria. The proposed method provides a simple, rapid, and cost-effective technique for producing AgNP nanocomposites.

## Data Availability

The data presented in this study is available upon request from the corresponding author.

## Supplementary Materials

The following supporting information can be downloaded at: https://www.mdpi.com/article/10.3390/nano13111784/s1, Figure S1: Extinction spectra of Ag/SU-8 nanocomposites fabricated on the SU-8 film without the incorporation of additional PI, demonstrating the effects of 1 and 3 cycles of photoreduction.
