# Supplementary material for: Synthesis of Metal/SU-8 Nanocomposites through Photoreduction on SU-8 Substrates
Source: Nanomaterials (Basel). 2023 Jun 1;13(11):1784. doi: 10.3390/nano13111784 (PMC10254310; doi:10.3390/nano13111784)
Supplement: Supplementary file 1 [file nanomaterials-13-01784-s001.zip › nanomaterials-2424710-supplementary.pdf]

# Supporting Information

## Synthesis of metal/SU-8 Nanocomposites through Photoreduction on SU-8 Substrates

Yan-Jun Huang [1], Wen-Huei Chang [2,*], Yi-Jui Chen [1] and Chun-Hung Lin [1,*]

[1] Department of Photonics, National Cheng Kung University, Tainan 70101, Taiwan

[2] Department of Applied Chemistry, National Pingtung University, Pingtung 90003, Taiwan

*Correspondence: whchang@mail.nptu.edu.tw (W.-H.C.);
chlin@ncku.edu.tw (C.-H.L.)

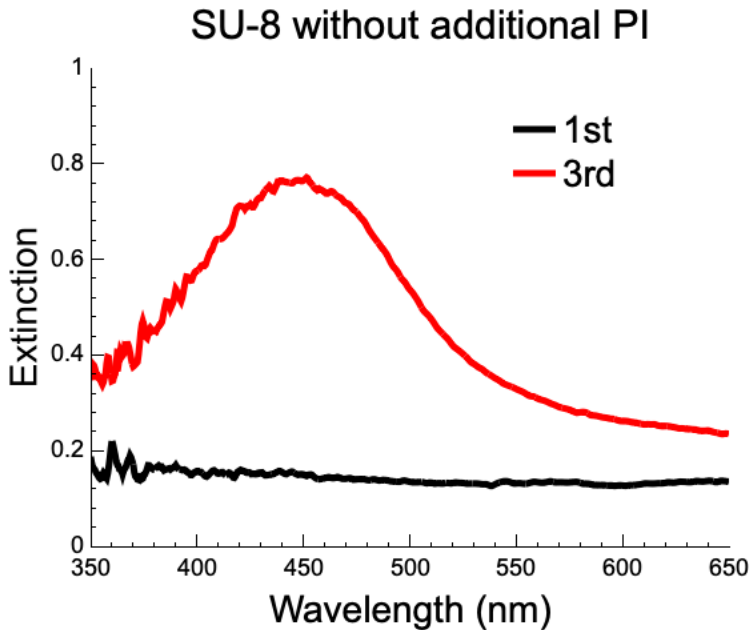


**Figure S1.** Extinction spectra of Ag/SU-8 nanocomposites fabricated on the SU-8 film without the incorporation of additional PI, demonstrating the effects of 1 and 3 cycles of photoreduction.
